# High-level accumulation of oleyl oleate in plant seed oil by abundant supply of oleic acid substrates to efficient wax ester synthesis enzymes

**DOI:** 10.1186/s13068-018-1057-4

**Published:** 2018-03-01

**Authors:** Dan Yu, Ellen Hornung, Tim Iven, Ivo Feussner

**Affiliations:** 1 Department of Plant Biochemistry, Albrecht-von-Haller-Institute for Plant Sciences, University of Goettingen, Justus-von-Liebig-Weg 11, 37077 Goettingen, Germany; 2 Department of Plant Biochemistry, Center for Molecular Biosciences (GZMB), University of Goettingen, Justus-von-Liebig-Weg 11, 37077 Goettingen, Germany; 3 Department of Plant Biochemistry, International Center for Advanced Studies of Energy Conversion (ICASEC), University of Goettingen, Justus-von-Liebig-Weg 11, 37077 Goettingen, Germany

**Keywords:** Wax esters, Fatty acyl reductase, Wax synthase, *Marinobacter aquaeolei*, *Acinetobactor baylyi*, *Mus musculus*, *Camelina sativa*, Jojoba, Fusion enzyme

## Abstract

**Background:**

Biotechnology enables the production of high-valued industrial feedstocks from plant seed oil. The plant-derived wax esters with long-chain monounsaturated acyl moieties, like oleyl oleate, have favorite properties for lubrication. For biosynthesis of wax esters using acyl-CoA substrates, expressions of a fatty acyl reductase (FAR) and a wax synthase (WS) in seeds are sufficient.

**Results:**

For optimization of the enzymatic activity and subcellular localization of wax ester synthesis enzymes, two fusion proteins were created, which showed wax ester-forming activities in *Saccharomyces cerevisiae*. To promote the formation of oleyl oleate in seed oil, WSs from *Acinetobactor baylyi* (*Ab*WSD1) and *Marinobacter aquaeolei* (*Ma*WS2), as well as the two created fusion proteins were tested in Arabidopsis to evaluate their abilities and substrate preference for wax ester production. The tested seven enzyme combinations resulted in different yields and compositions of wax esters. Expression of a FAR of *Marinobacter aquaeolei* (*Ma*FAR) with *Ab*WSD1 or *Ma*WS2 led to a high incorporation of C_18_ substrates in wax esters. The *Ma*FAR/TM*Mm*AWAT2-*Ab*WSD1 combination resulted in the incorporation of more C_18:1_ alcohol and C_18:0_ acyl moieties into wax esters compared with *Ma*FAR/*Ab*WSD1. The fusion protein of a WS from *Simmondsia chinensis* (*Sc*WS) with MaFAR exhibited higher specificity toward C_20:1_ substrates in preference to C_18:1_ substrates. Expression of *Ma*FAR/*Ab*WSD1 in the Arabidopsis *fad2 fae1* double mutant resulted in the accumulation of oleyl oleate (18:1/18:1) in up to 62 mol% of total wax esters in seed oil, which was much higher than the 15 mol% reached by *Ma*FAR/*Ab*WSD1 in Arabidopsis Col-0 background. In order to increase the level of oleyl oleate in seed oil of *Camelina*, lines expressing *Ma*FAR/*Sc*WS were crossed with a transgenic high oleate line. The resulting plants accumulated up to >40 mg g seed^−1^ of wax esters, containing 27–34 mol% oleyl oleate.

**Conclusions:**

The overall yields and the compositions of wax esters can be strongly affected by the availability of acyl-CoA substrates and to a lesser extent, by the characteristics of wax ester synthesis enzymes. For synthesis of oleyl oleate in plant seed oil, appropriate wax ester synthesis enzymes with high catalytic efficiency and desired substrate specificity should be expressed in plant cells; meanwhile, high levels of oleic acid-derived substrates need to be supplied to these enzymes by modifying the fatty acid profile of developing seeds.

**Electronic supplementary material:**

The online version of this article (10.1186/s13068-018-1057-4) contains supplementary material, which is available to authorized users.

## Background

In recent years, the production of high-value chemicals from plant oil has drawn an increasing attention, due to the enhancing requirement of low-priced, environmentally friendly, and renewable industrial feedstocks [1–3]. The development of modern biotechnological tools and the growing knowledge of plant lipid metabolism have given a solid foundation for producing industrial commodities like wax esters from plants. Wax esters are a group of highly hydrophobic neutral lipids existing in various organisms [4–7]. Due to the chemical and physical properties of wax esters, they find diverse commercial applications, such as in production of cosmetics, surface coatings, and lubricants. Wax esters used as lubricants should have a low melting temperature and a high oxidation stability. Hence, wax esters species which consist of monounsaturated alcohols and acids with medium or long carbon chains, such as oleyl oleate, are known to have favorite properties for lubrication.

The availability of wax esters for industrial applications is limited, although they are commonly found in nature. Spermaceti oil mainly consists of oleyl oleate and was a popular lubricant [8]. After the ban of whale hunting, a suitable replacement [6] of spermaceti wax, not deriving from fossil resources, could not be identified for a long time, until the desert shrub jojoba (*Simmondsia chinensis*) was found to accumulate wax esters instead of triacylglycerols (TAGs) as storage lipids in seeds [7]. However, jojoba oil is composed of very long-chain wax esters (C_34_–C_48_) with one double bond in each moiety, which are unfavorable in cold conditions [7, 9]. In addition, jojoba is not suitable for large-scale cultivation in moderate temperate zones, so that jojoba oil is an expensive material.

Wax esters derived from plant oil can be an alternative resource for commercial applications [1, 3]. To establish a biosynthesis pathway of wax esters in plants, only two enzymes are needed. First, a fatty acyl-CoA reductase (FAR) provides alcohol substrates for wax ester biosynthesis by reducing a fatty acyl-CoA or a fatty acyl-ACP to a primary fatty alcohol using NADPH as the reductant [10, 11]. The second enzyme is a wax synthase (WS) that catalyzes the esterification of a fatty alcohol with a fatty acyl-CoA or a fatty acyl-ACP to yield a wax ester molecule [12, 13].

The heterologous biosynthesis of wax esters in plant seeds started from the introduction of a wax ester synthesis pathway in the model plant Arabidopsis. Seed TAGs were replaced by jojoba oil-like wax esters, when the two necessary enzymes for wax synthesis from jojoba (*Sc*FAR and *Sc*WS) were coexpressed in Arabidopsis [12]. Recently, different combinations of FARs and WSs that had various origins were tested in Arabidopsis for wax ester production. These enzyme combinations resulted in the accumulation of wax esters ranging from 17 mg g seed^−1^ to >100 mg g seed^−1^, with the highest yield achieved by coexpression of a FAR from *M. aquaeolei* (*Ma*FAR) with *Sc*WS [14, 15]. The wax ester compositions were affected by the substrate preference of different wax synthesis enzymes. The combination of mouse enzymes (*Mm*FAR/*Mm*WS) led to the production of saturated alcohols esterified to polyunsaturated acyl-CoAs, while *Ma*FAR/*Sc*WS showed high specificity to monounsaturated substrates [15]. In addition, 60 mol% oleyl oleate of total wax esters was accumulated by *Mm*FAR/*Mm*WS and *Ma*FAR/*Sc*WS combinations in the high oleic Arabidopsis *fad2 fae1* double mutant [14, 15].

The wax ester biosynthesis was subsequently established in an oilseed crop *Camelina*. Having an oil content of 30–40% of seed weight [16, 17] and many considerable agronomic traits [18–21]. *C. sativa* is attracting more and more attention as a potential platform of metabolic engineering for unusual industrial oils. *Camelina* can be simply transformed by *Agrobacteria tumefaciens*-mediated floral dip infiltration [22]. Lately, the whole genome [23] and the seed transcriptome [24–28] of *Camelina* have been made available. In a recent study, >40 mg g seed^−1^ of wax esters was accumulated in *Camelina* seed oil by the *Ma*FAR/*Sc*WS combination; however, the levels of oleyl oleate were below 5% of total wax esters in *Camelina* transgenic lines [15].

In order to further improve the formation of oleyl oleate in seed oil, optimization of wax ester synthesis enzymes and modification of wax ester composition by adjusting acyl-CoA pools were performed in this study. WSs with a higher potential for the desired specificity were tested by heterologous expression in yeast and Arabidopsis, and fusion proteins were created for altered subcellular localization of the enzymes to improve their activities. In total, seven combinations of wax ester synthesis enzymes were expressed in Arabidopsis to evaluate their abilities for oleyl oleate production. In addition, a suitable enzyme combination was expressed in a transgenic high oleate *Camelina* line, and this led to the production of high levels of oleyl oleate in seed oil.

## Results

### Expression of fusion proteins in *S. cerevisiae* yielded active enzymes

*Sc*WS is most likely localized in the ER membrane, while *Ma*FAR is a cytosolic protein. Even though coexpression of *Ma*FAR with *Sc*WS in Arabidopsis and *Camelina* resulted in high yields of wax esters in seed oil [15], colocalization of *Ma*FAR with *Sc*WS in the same membrane may increase wax ester production. Therefore, a *Sc*WS-*Ma*FAR fusion protein was generated by fusing *Ma*FAR to the C-terminal end of *Sc*WS. The ability of *Sc*WS-*Ma*FAR to produce wax esters was tested by heterologous expression in *S. cerevisiae*. Fatty alcohols and wax esters do not naturally accumulate in *S. cerevisiae*. Some cultures were therefore additionally supplemented with fatty alcohols to test WS activity independently of FAR activity. While as expected the coexpression of the single enzymes *Ma*FAR/*Sc*WS resulted in production of both fatty alcohols and wax esters, the *Sc*WS-*Ma*FAR fusion protein produced fatty alcohols and wax esters as well. These results show that the *Sc*WS-*Ma*FAR fusion protein has both FAR and WS activities (Additional file 1: Figure S1a).

**Fig. 1:**
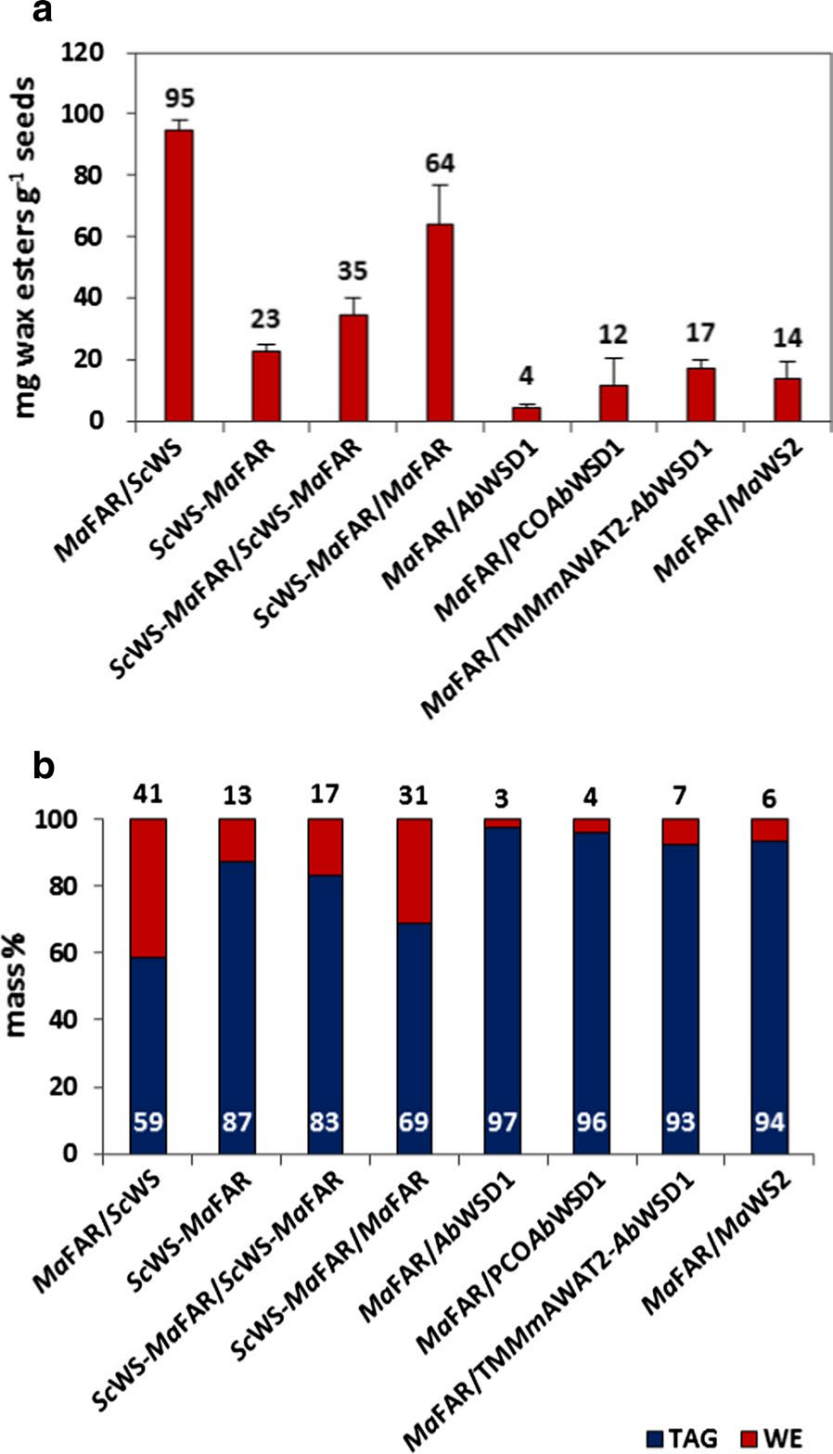
Quantification of wax esters in seeds of Arabidopsis. Transgenic lines transformed with *Ma*FAR/*Sc*WS, *Sc*WS-*Ma*FAR, *Sc*WS-*Ma*FAR/*Sc*WS-*Ma*FAR, *Sc*WS-*Ma*FAR/*Ma*FAR, *Ma*FAR/*Ab*WSD1, *Ma*FAR/PCO*Ab*WSD1, *Ma*FAR/TM*Mm*AWAT2-*Ab*WSD1, and *Ma*FAR/*Ma*WS2 are shown. **a** Absolute quantification of wax esters in mg g seed^−1^ by GC-FID. The data shown represent an average of three individual transgenic lines for each enzyme combination with two extraction replicates for each individual line (+SD). **b** The relative quantification of total neutral lipids (WE, wax ester; TAG, triacylglycerol) in mass % are calculated according to the absolute quantification of each lipid class. The data shown represent an average of three individual transgenic lines for each enzyme combination with two extraction replicates. Raw data are provided as Additional file 2: Table S1

*Ab*WSD1 is a bifunctional WS/DGAT enzyme from *A. baylyi* ADP1, showing high preference for C_18_ substrates [13, 29, 30]. In this study, coexpression of *Ab*WSD1 with *Ma*FAR resulted in low amounts of wax esters in Arabidopsis seeds (Fig. 1, *Ab*WSD1/*Ma*FAR). Therefore, the first 20 amino acids of *Ab*WSD1 were optimized for plant codon usage (PCO*Ab*WSD1) as described previously [31], to increase its expression level in plant cells. In addition, a TM*Mm*AWAT2-*Ab*WSD1 fusion protein was generated by fusing two transmembrane domains (first 60 AA) of *Mm*AWAT2 to the N-terminal end of *Ab*WSD1, for a potential enhancement of enzymatic activity by targeting the enzyme to the ER membrane [32]. To test their WS activity, PCO*Ab*WSD1 and TM*Mm*AWAT2-*Ab*WSD1 were expressed in *S. cerevisiae*. When feeding yeast cells with fatty alcohol, *Mm*AWAT2 and PCO*Ab*WSD1 only produced wax esters. The TM*Mm*AWAT2-*Ab*WSD1 fusion protein synthesized both wax esters and TAGs in *S. cerevisiae*, showing its activity as a bifunctional WS/DGAT enzyme (Additional file 1: Figure S1b).

### Different enzyme combinations produced varying amounts of wax esters in seeds of Arabidopsis

**Fig. 2:**
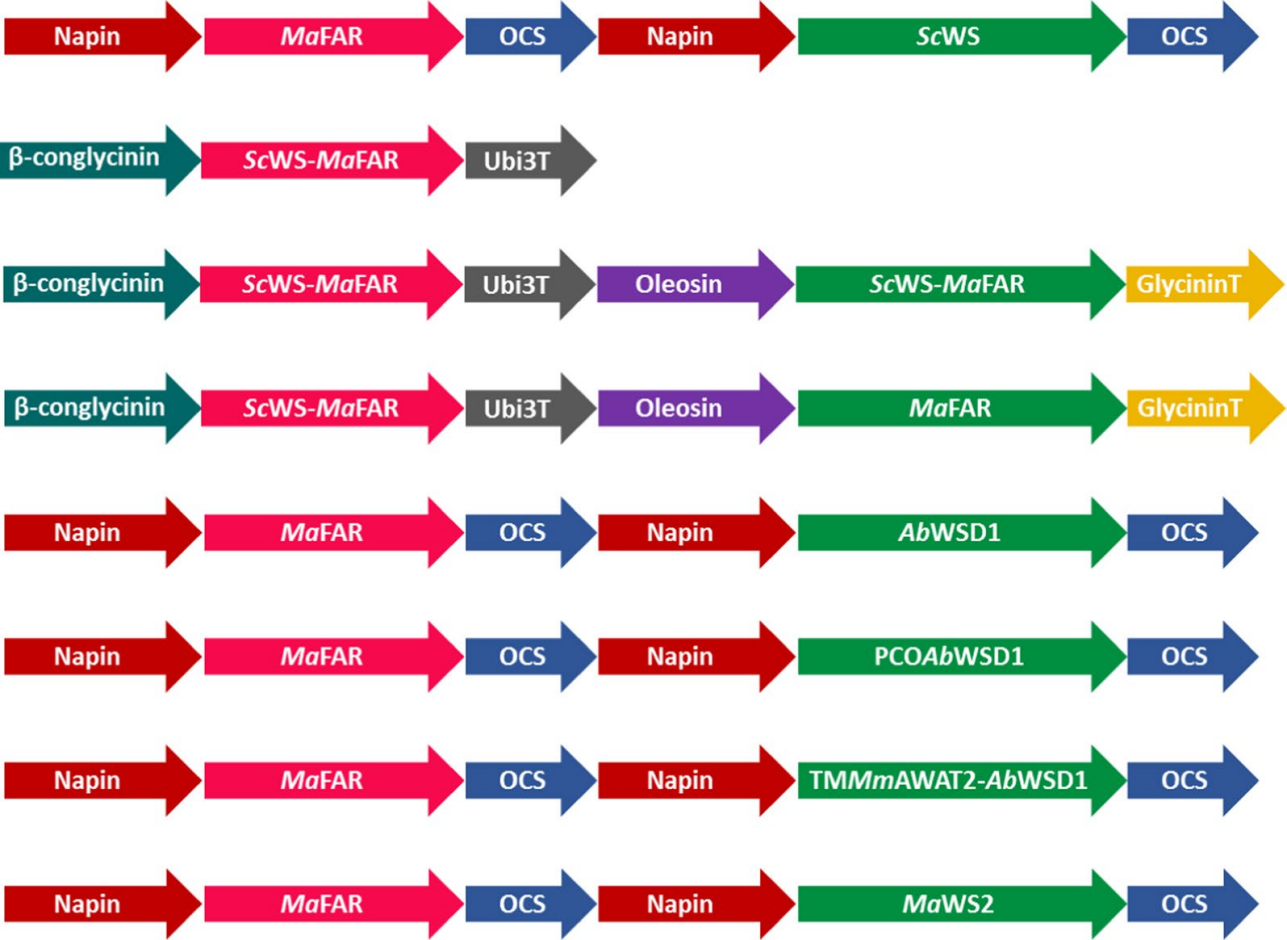
DNA constructs used for seed-specific expression of different combinations of wax ester synthesis enzymes

To obtain high content of oleyl oleate from plant-derived wax esters, two bacterial WSs, *Ab*WSD1 from *A. baylyi* and *Ma*WS2 from *M. aquaeolei*, as well as the optimized *Ab*WSD1 versions were coexpressed with *Ma*FAR. In addition, the transgene for the *Sc*WS-*Ma*FAR fusion protein was either transferred as single copy, double copies or together with *Ma*FAR to increase the abundance of wax ester forming enzymes and the supply of fatty alcohols. In total, seven enzyme combinations were expressed in seeds of Arabidopsis: *Sc*WS-*Ma*FAR, *Sc*WS-*Ma*FAR/*Sc*WS-*Ma*FAR, *Sc*WS-*Ma*FAR/*Ma*FAR, *Ma*FAR/*Ab*WSD1, *Ma*FAR/PCO*Ab*WSD1, *Ma*FAR/TM*Mm*AWAT2-*Ab*WSD1 and *Ma*FAR/*Ma*WS2 (Fig. 2).

After screening at least 30 heterozygous T_2_ lines for each combination, the best three performing lines of each combination were selected for quantification of wax esters and TAGs. The *Sc*WS-*Ma*FAR lines produced an average of 23 mg g seed^−1^ of wax esters, and the second copy of *Sc*WS-*Ma*FAR further increased the yield up to 35 mg g seed^−1^ (Fig. 1a). The *Sc*WS-*Ma*FAR/*Ma*FAR coexpression led to the accumulation of wax esters up to 64 mg g seed^−1^, accounting for 31% of total neutral lipids (Fig. 1b). The *Ma*FAR/*Ma*WS2 combination produced wax esters up to 14 mg g seed^−1^, accounting for 6% of total neutral lipids (Fig. 1). However, very low yield of wax esters (4 mg g seed^−1^) was achieved by *Ma*FAR/*Ab*WSD1, while the *Ma*FAR/PCO*Ab*WSD1 and *Ma*FAR/TM*Mm*AWAT2-*Ab*WSD1 combinations enabled to increase the yields of wax esters to 12 and 17 mg g seed^−1^, respectively (Fig. 1a). Overall, lower yields of wax esters were reached by the seven tested enzyme combinations in Arabidopsis seed oil, in comparison to *Ma*FAR/*Sc*WS lines obtained previously (Fig. 1) [15].

### Different enzyme combinations showed diverse substrate specificities

**Fig. 3:**
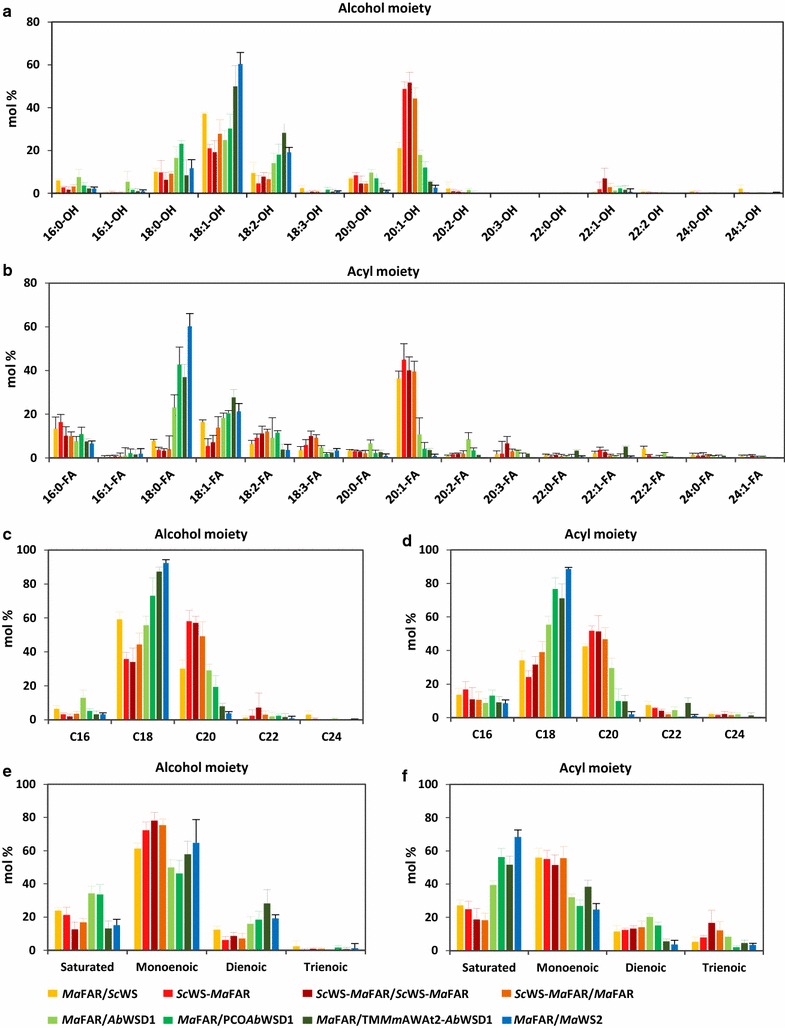
Alcohol and acyl moieties of wax esters in seeds of Arabidopsis. Transgenic lines transformed with *Ma*FAR/*Sc*WS (yellow bar), *Sc*WS-*Ma*FAR (red bar), *Sc*WS-*Ma*FAR/*Sc*WS-*Ma*FAR (dark red bar), *Sc*WS-*Ma*FAR/*Ma*FAR (orange bar), *Ma*FAR/*Ab*WSD1 (light green bar), *Ma*FAR/PCO*Ab*WSD1 (green bar), *Ma*FAR/TM*Mm*AWAT2-*Ab*WSD1 (dark green bar) and *Ma*FAR/*Ma*WS2 (blue bar) are shown. **a** Relative abundance of alcohol moieties in mol%. **b** Relative abundance of acyl moieties in mol%. **c** Alcohol moiety calculated by total carbon number. **d** Acyl moiety calculated by total carbon number. **e** Alcohol moiety calculated by desaturation degree. **f** Acyl moiety calculated by desaturation degree. The data shown represent an average of three individual transgenic lines for each enzyme combination with two extraction replicates. Raw data are provided as Additional file 2: Table S1

The compositions of wax esters produced by the seven enzyme combinations were obviously diverse. The *Ma*FAR/*Sc*WS coexpression mainly incorporated 18:1-OH (40 mol%) and 20:1-FA (38 mol%) into wax esters (Fig. 3). Differently, the three combinations expressing *Sc*WS-*Ma*FAR fusion protein predominantly utilized 20:1-OH that accounted for 45–52 mol% of total fatty alcohol moieties, meanwhile a lower abundance of 18:1-OH (20–28 mol%) was observed (Fig. 3a). Furthermore, in comparison to *Ma*FAR/*Sc*WS, a lower level of 18:1-FA and a higher level of 20:1-FA were found in wax esters produced by the *Sc*WS-*Ma*FAR (Fig. 3b). *Ab*WSD1 and *Ma*WS2 were reported to have high preference for C_18_ substrates [29, 30, 33]. As expected, *Ma*FAR/*Ab*WSD1 and *Ma*FAR/PCO*Ab*WSD1 combinations showed similar substrate preference, incorporating high levels of 18:1-OH and 18:0-FA into wax esters. Interestingly, the *Ma*FAR/TM*Mm*AWAT2-*Ab*WSD1 utilized higher levels of C_18:1_ substrates and lower levels of C_20:1_ substrates, compared with *Ma*FAR/*Ab*WSD1 and *Ma*FAR/PCO*Ab*WSD1 (Fig. 3). The *Ma*FAR/*Ma*WS2 combination predominantly incorporated C_18:1_ at fatty alcohol moiety (Fig. 3a) and utilized C_18:0_ acyl moiety for wax ester production (Fig. 3b).

Further analysis of the acquired data showed different specificities of the seven enzyme combinations regarding chain length and saturation degree of alcohol and acyl moieties (Fig. 3c–f). As in previous research, *Ma*FAR/*Sc*WS coexpression revealed a dominant utilization of C_18_ alcohols (around 60 mol%) for wax ester biosynthesis; however, the length of alcohol chain was obviously shifted from C_18_ to C_20_ by the *Sc*WS-*Ma*FAR fusion protein (15, Fig. 3c). Furthermore, the three enzyme combinations expressing *Sc*WS-*Ma*FAR fusion protein also showed a high incorporation of C_20_ acyl moieties (>50 mol%) into wax esters (Fig. 3d). The *Ma*FAR/*Ab*WSD1 and *Ma*FAR/PCO*Ab*WSD1 combinations predominantly use alcohols and acyl substrates in C_18_ chain length, the latter one showing higher selectivity for C_18_ chain length (Fig. 3c, d). Interestingly, *Ma*FAR/TM*Mm*AWAT2-*Ab*WSD1 showed with >80 mol% incorporation of C_18_ alcohols, even higher preference than *Ma*FAR/*Ab*WSD1 for these substrates (Fig. 3c). The *Ma*FAR/*Ma*WS2 combination revealed the highest incorporation of C_18_ substrates into wax esters, with 90 mol% C_18_ alcohol moieties and >80 mol% C_18_ acyl moieties (Fig. 3c, d).

With regard to the saturation degree of alcohol and acyl moieties, the three combinations with *Sc*WS-*Ma*FAR incorporated around 80 mol% monoenoic alcohols, higher than the 60 mol% in *Ma*FAR/*Sc*WS (Fig. 3e), while similar levels of monoenoic acyl moieties (>50 mol%) were observed in both *Ma*FAR/*Sc*WS coexpression and *Sc*WS-*Ma*FAR lines (Fig. 3f). Comparatively, *Ma*FAR/*Ab*WSD1 and *Ma*FAR/PCO*Ab*WSD1 combinations showed lower specificity to monoenoic substrates, using around 50 mol% monounsaturated alcohols and 40 mol% monounsaturated acyl substrates. Instead, these two combinations preferred saturated and dienoic alcohols, as well as saturated acyl substrates for wax ester biosynthesis (Fig. 3e, f). Whereas, while *Ma*FAR/TM*Mm*AWAT2-*Ab*WSD1 displayed similar preference to monoenoic substrates, it tends to use higher levels of unsaturated alcohols instead of saturated alcohols, compared with *Ma*FAR/*Ab*WSD1. Moreover, *Ma*FAR/*Ma*WS2 dominantly incorporated monoenoic substrates (>60 mol%) at the alcohol position and saturated ones (around 70 mol%) at the acyl position (Fig. 3e, f).

### Specific production of oleyl oleate was achieved by expression of *Ma*FAR/*Ab*WSD1 in Arabidopsis *fad2 fae1* double mutant

**Fig. 4:**
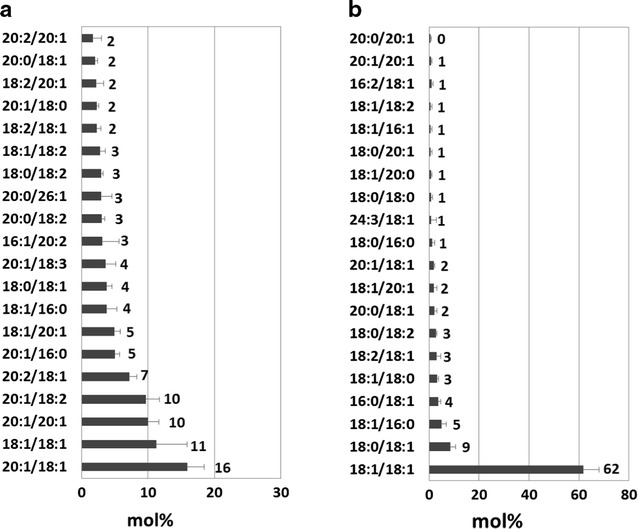
Molecular species of wax esters in seeds of Arabidopsis transformed with *Ma*FAR/*Ab*WSD1. **a** In Col-0 background. **b** In *fad2 fae1* double mutant. Wax ester molecular species were determined by nano-ESI–MS/MS. The relative abundance of the top twenty wax ester molecular species (alcohol moiety/acyl moiety) in mol% are shown. The data shown represent an average of ten individual T2 heterozygous transgenic lines. Raw data are provided as Additional file 3: Table S2

The molecular species of wax esters produced by *Ma*FAR/*Ab*WSD1 Arabidopsis seeds were measured by ESI–MS/MS. The *Ma*FAR/*Ab*WSD1 combination in Arabidopsis Col-0 background led to accumulation of 4 mg g seed^−1^ of wax esters (Fig. 1a) and 11 mol% 18:1/18:1 in all wax ester species, which was similar to MaFAR/ScWS (10 mol%) and higher than those of other previously studied enzyme combinations (Fig. 4a) [15]. The most abundant wax ester species 20:1/18:1 accumulated by *Ma*FAR/*Ab*WSD1 still accounted for 16 mol% of total wax esters, and additional accumulation of 20:1/20:1 (10 mol%), 20:1/18:2 (10 mol%) and 20:2/18:1 (7 mol%) were observed (Fig. 4a). Therefore, the substrate preference of *Ma*FAR/*Ab*WSD1 is suitable for the formation of oleyl oleate, but specific production of oleyl oleate to a high level was not reached by simply expressing enzymes with a higher substrate specificity.

It was shown that the profile of fatty acyl-CoAs for wax ester biosynthesis significantly influence molecular species of wax esters [14]. For specific accumulation of oleyl oleate, *Ma*FAR/*Ab*WSD1 was therefore also expressed in the Arabidopsis *fad2 fae1* double mutant that is enriched in oleic acid in seed oil [34]. This led to accumulation of 5 mg g seed^−1^ of wax esters and up to 62 mol% 18:1/18:1 of total wax esters accumulated by expressing *Ma*FAR/*Ab*WSD1 in this high oleate background, resulting in a similar level of oleyl oleate that was reached by the previously studied enzyme combinations in the same background (Fig. 4b) [14, 15].

### Crossing *Ma*FAR/*Sc*WS lines with a high oleate line led to high amount of oleyl oleate in seeds of *Camelina*

**Fig. 5:**
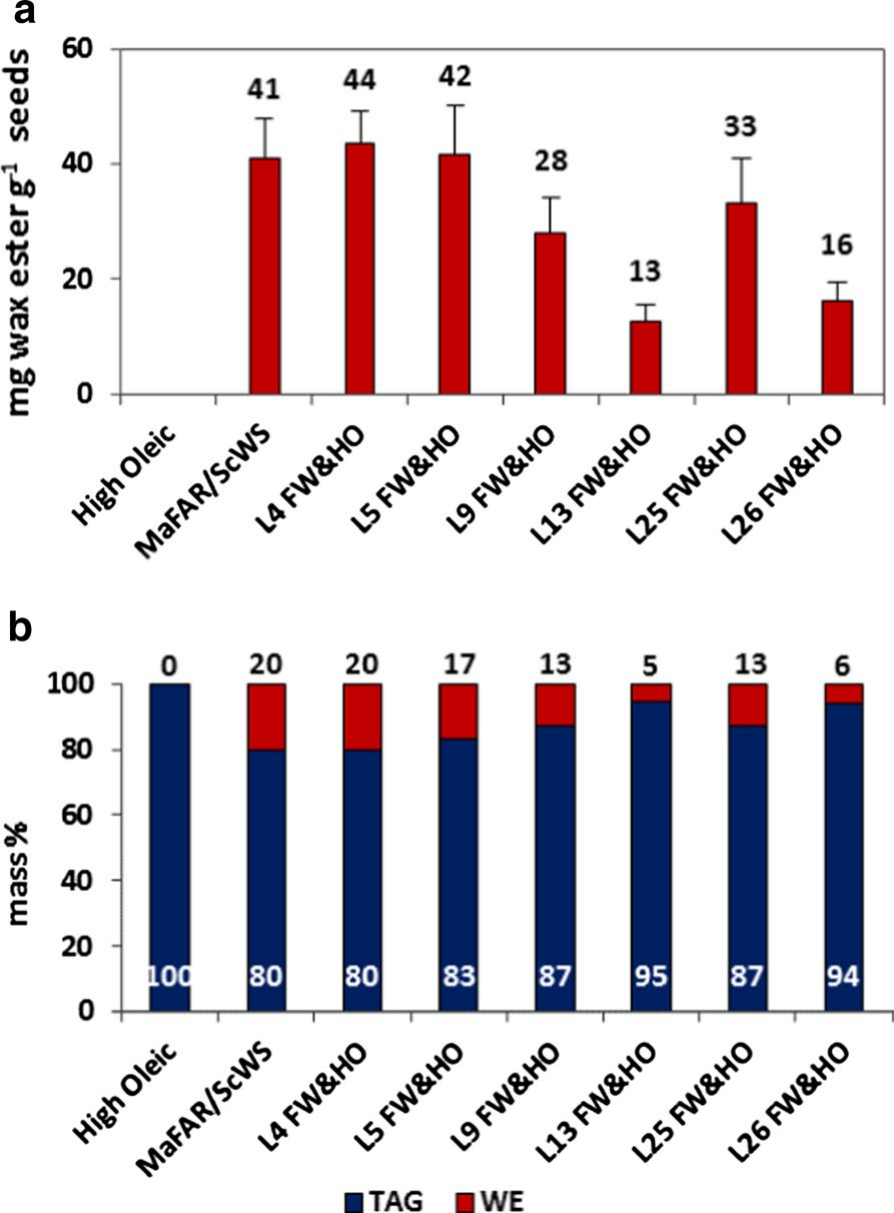
Quantification of wax esters in seeds of *Camelina*. Transgenic lines containing high levels of oleic acid (High Oleic), with *Ma*FAR/*Sc*WS, and six crosses of *Ma*FAR/*Sc*WS lines with High Oleic line (FW&HO) were shown. **a** Absolute quantification of wax esters in mg g seed^−1^. The data shown represent an average of three individual transgenic lines for each independent cross with two extraction replicates for each individual line (+SD). **b** The relative quantification of total neutral lipids (WE, wax ester; TAG, triacylglycerol) in mass % are calculated according to the absolute quantification of each lipid class. The data shown represent an average of three individual transgenic lines for each independent cross with two extraction replicates for each individual line. Raw data are provided as Additional file 4: Table S3

Among all tested enzyme combinations in previous and current studies, the *Ma*FAR/*Sc*WS combination led to limited accumulation of oleyl oleate (4.7 mol%), but the highest yield of wax esters (around 40 mg g seed^−1^) in seeds of Arabidopsis and *Camelina* (Fig. 1) [14, 15]. A high oleate (HO) *Camelina* line was kindly provided by Prof. Cahoon [24], which was generated via a RNA_i_ approach, using FAD2/FAE1 RNA_i_ sequences from *Camelina* and FAD3 RNA_i_ sequence from Arabidopsis and contains a favorable fatty acid profile for the formation of oleyl oleate. Therefore, six individual *Ma*FAR/*Sc*WS *Camelina* lines with relatively high wax ester contents were crossed with the HO-line, resulting in six independent *Ma*FAR/*Sc*WS and High Oleic crosses (*Ma*FAR/*Sc*WS-HO). The yields of wax esters in seeds of these six *Ma*FAR/*Sc*WS-HO ranged from 13 to 40 mg g seed^−1^, accounting for 5–20% of total neutral lipids. Among them, two crosses (L4 and L5) resulted in the highest wax ester amounts exceeding 40 mg g seed^−1^ (Fig. 5).

**Fig. 6:**
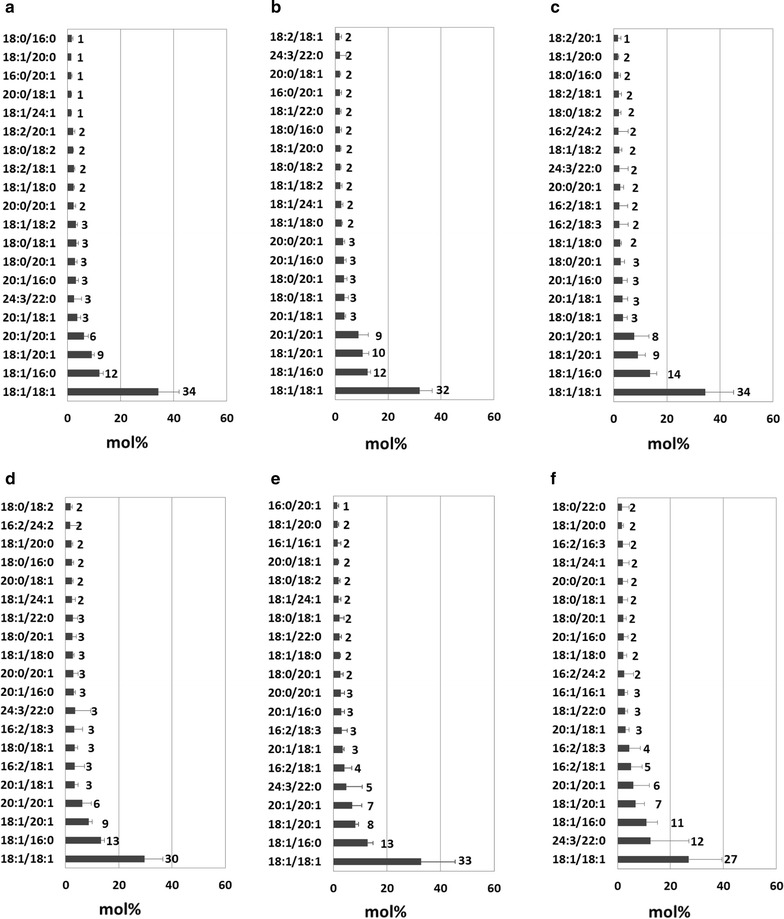
Molecular species of wax esters in seeds of six *Camelina Ma*FAR/*Sc*WS & High Oleic crosses. **a** L4 *Ma*FAR/*Sc*WS & High Oleic cross; **b** L5 *Ma*FAR/*Sc*WS & High Oleic cross; **c** L9 *Ma*FAR/*Sc*WS & High Oleic cross; **d** L13 *Ma*FAR/*Sc*WS & High Oleic cross; **e** L25 *Ma*FAR/*Sc*WS & High Oleic cross; **f** L26 *Ma*FAR/*Sc*WS & High Oleic cross. Wax ester compositions were determined by nano-ESI–MS/MS. The relative abundance of the top twenty wax ester molecular species (alcohol moiety/acyl moiety) are shown. The data shown represent an average of seven individual heterozygous transgenic lines resulting from the six independent crosses with two extraction replicates for each individual line. Raw data are provided as Additional file 5: Table S4

All six *Ma*FAR/*Sc*WS-HO resulted in a much higher accumulation of 18:1/18:1 wax esters, compared with the original *Ma*FAR/*Sc*WS lines (Fig. 6) [15]. Importantly, the most abundant wax ester species is 18:1/18:1 for all produced *Ma*FAR/*Sc*WS-HO, with the range from 27% for L26 *Ma*FAR/*Sc*WS-HO to 34% for L4 and L9 *Ma*FAR/*Sc*WS-HO (Fig. 6a, c, f). Some individual lines of *Ma*FAR/*Sc*WS-HO even accumulated up to 45 mol% 18:1/18:1 of total wax esters (Additional file 5: Table S4). *Ma*FAR/*Sc*WS accumulated large amounts of very long-chain wax esters (C_38_–C_40_), with 17.7 mol% 18:1/20:1 and 10 mol% 20:1/20:1 in *Camelina* seed oil [15]. However, the levels of 18:1/20:1 decreased to 7 mol–10 mol%, and the levels of 20:1/20:1 decreased to 7 mol% in wax esters produced by *Ma*FAR/*Sc*WS-HO; in contrary, more wax esters with shorter chain length (C_34_–C_36_) were produced, with the levels of 18:1/16:0 increased to around 12 mol% (Fig. 6).

Overall, *Ma*FAR/*Sc*WS-HO led to an increased accumulation of 18:1/18:1 up to 34 mol% of total wax esters. Meanwhile, the total yields of wax esters in seeds of *Camelina* were not negatively affected by crossing. In addition, some seedlings of *Ma*FAR/*Sc*WS-HO were also delayed in germination and had white cotyledons, as previously observed for *Ma*FAR/*Sc*WS lines (Additional file 6: Figure S2).

## Discussion

In the present study, we followed three strategies to improve the formation of oleyl oleate in plant seed oil: (i) optimization of wax ester synthesis enzymes, (ii) identification of enzyme combinations for higher activities and appropriate substrate specificities, (iii) modification of fatty acyl substrate pool for wax synthesis pathway.

In case of the first approach of optimizing available wax ester synthesis enzymes, two additional WS enzymes and three enzyme fusions were tested. Overall, we were able to identify three enzymes with a better specificity than *Sc*WS: *Ma*WS2, TM*Mm*AWAT2-*Ab*WSD1 and PCO*Ab*WSD1 (Fig. 3). However, the gained specificity was always on the expense of reduced wax yields (Fig. 1). On a first glance, this may be either due to lower enzyme amounts or activities. One explanation for low enzyme amounts may be suboptimal codons of the used cDNA and indeed the codon usage values of *MaFAR* and *ScWS* indicate low protein abundance which may even affect the long *Sc*WS-*Ma*FAR fusion protein in comparison to the single proteins (Additional file 7: Figure S3). In addition, this may explain the lower wax ester biosynthesis rate in *S. cerevisiae* (Additional file 1: Figure S1). The TM*Mm*AWAT2-*Ab*WSD1 fusion protein seemed to produce more wax esters than PCO*Ab*WSD1 upon expression in *S. cerevisiae* (Additional file 1: Figure S1), but the differences in wax ester amount could not be linked conclusively to higher activity of TM*Mm*AWAT2-*Ab*WSD1 as it could also be due to higher protein abundance in *S. cerevisiae*. Future work will determine whether these differences are due to protein stability or increased enzyme activity.

To follow the second approach, seven enzyme combinations were tested. All three combinations expressing the *Sc*WS-*Ma*FAR fusion protein produced less wax esters compared with *Ma*FAR/*Sc*WS coexpression (Fig. 1). The fact that two copies of *Sc*WS-*Ma*FAR showed higher amounts of wax esters than only one may suggest that the abundance of the fusion protein is lower when it is expressed under the control of the soybean β-conglycinin promoter or oleosin promoter than under the control of the napin promoter used in previous experiments (Fig. 2). Interestingly, the additional expression of another copy of *Ma*FAR, also under the control of the oleosin promoter, displayed an increase in wax ester production to almost three times in comparison to expression of fusion-protein alone. However, any further comparison with the *Ma*FAR/*Sc*WS construct is difficult since this was expressed under the control of the napin promoter.

To the best of our knowledge, two bacterial WSs were expressed in plant seeds for the first time. When *Ma*FAR was coexpressed with *Ab*WSD1 or *Ma*WS2 in Arabidopsis, only low amounts of wax esters were accumulated in the seed oil of Arabidopsis (Fig. 1). Comparatively, the combinations of vertebrate-type and plant-type enzymes led to higher yields of wax esters, such as 33 mg g seed^−1^ for Oleo3:mCherry:*Mm*FAR1Δc/Oleo3:EYFP:*Mm*WS and 100 mg g seed^−1^ for *Ma*FAR/*Sc*WS [14, 15]. The low yields caused by the *Ma*FAR/*Ab*WSD1 and *Ma*FAR/*Ma*WS2 combinations can only be attributed to the WS, and not to a low supply of fatty alcohols, as in previous experiments coexpression of other WS with *Ma*FAR led to high yields of wax esters. Reasons for this might be on one hand, that the catalytic activities of these bacterial WSs could be inhibited in plant cells due to the distinct intercellular environment compared to its original host cells. On the other hand, in bacteria substrates for WSs might also be fatty acyl-ACPs instead of fatty acyl-CoAs [13]; hence, as cytosolic enzymes, they are not able to access the putatively more favorable fatty acyl-ACP substrates localized in plastids.

In the case of *Ab*WSD1, the abundance of the WS might also play a role in low production of wax esters, as the codon optimized *Ab*WSD1 (PCO*Ab*WSD1) as well as the fusion protein with the eukaryotic transmembrane domain of mouse wax ester synthase (TM*Mm*AWAT2-*Ab*WSD1) resulted in a three to four times higher accumulation of wax esters (Fig. 1). In addition, the sequestration of produced wax esters into lipid bodies could be affected by a cytosolic WS associated with the ER membrane (TM*Mm*AWAT2-*Ab*WSD1). It is a common feature of other enzymes involved in the biosynthesis of neutral lipids to be localized to the ER, as there lipid bodies are formed.

Among the seven enzyme combinations, the highest preference for C_18_ substrates was observed for *Ma*FAR/*Ma*WS2. This combination also exhibited a trend for high incorporation of monounsaturated alcohols but a high preference for saturated acyl substrates (Fig. 3). This specificity of a WS in regard to the saturation degree of acyl-CoAs would be a negative factor for the formation of oleyl oleate.

In vitro *Ab*WSD1 can accept C_2_–C_30_ substrates with the highest activity against the C_18:1_ alcohol and C_16:0_ acyl-CoA [29]. This preference was not so clearly displayed upon expression of *Ma*FAR/*Ab*WSD1 in Arabidopsis. Here the WS incorporated mainly C_18_ alcohols and acyl substrates into wax esters, followed by C_20_ alcohols and acyl substrates (Fig. 3). Only small amounts of C_16_ moieties were detected in the wax esters probably because these fatty acyl chains are of low abundance. However, the profile of molecular species of wax esters produced by *Ma*FAR/*Ab*WSD1 showed that the most abundant species contained a C_20:1_ alcohol (Fig. 4a). In addition, the subsequent molecular species with the exception of the second most abundant one also comprise a C_20:1_ alcohol (Fig. 4a), while C_18_ acyl chains were prevailing in the most abundant species. These results indicate a high specificity of *Ab*WSD1 to C_20:1_ alcohol that was not tested in previous studies [29, 30].

Interestingly, the *Ma*FAR/TM*Mm*AWAT2-*Ab*WSD1 combination showed a higher incorporation of C_18_ substrates compared with *Ma*FAR/*Ab*WSD1 (Fig. 3c, d), showing that the specificity of TM*Mm*AWAT2-*Ab*WSD1 fusion protein was different from its original enzyme. This is probably because *Mm*AWAT2 also has a strong preference for C_18_ substrates, and its first two transmembrane domains may play an essential role on determining its substrate specificity [14, 32].

Moreover, the composition of the produced wax esters seems also to be affected by the subcellular localization of wax ester synthesis enzymes. In comparison to *Ma*FAR/*Sc*WS coexpression, the *Sc*WS-*Ma*FAR fusion protein led to an obvious alteration in compositions of wax esters (Fig. 3). While *Ma*FAR/*Sc*WS incorporated predominantely C_18:1_ alcohol into wax esters, the fusion protein preferred C_20:1_ alcohol. No significant differences were detected concerning the acyl substrate between the two enzyme combinations. This change in wax ester composition could be either because simply relocalization of the *Ma*FAR to the ER changes the access of the enzyme to very long-chain acyl-CoAs or more likely the fusion to *Sc*WS influences the availability of C_20_ acyl-CoAs. Also the catalytic activity of the fused *Ma*FAR could result in a preference for C_20_ acyl-CoAs.

A previous study showed that high levels of oleyl oleate (60 mol%) in Arabidopsis seeds were only achieved by expressing enzymes for wax ester synthesis in a high oleate background [14, 15], suggesting that modifying the profile of the acyl-CoA substrate pool is necessary for high accumulation of oleyl oleate. We show here, as third approach that the substrate pool has a higher influence than the enzyme specificity on the accumulation of oleyl oleate. Comparison of the molecular species of wax esters produced by *Ma*FAR/*Ab*WSD1 in Arabidopsis Col-0 background and in the high oleic *fad2 fae1* double mutant (Fig. 4) revealed that the relative amount of oleyl oleate increased from 11 mol% to >60 mol%. Meanwhile, the effect of a high oleate background also worked in *Camelina*, so that the six *Ma*FAR/*Sc*WS-HO resulted in >30 mol% oleyl oleate, which was significantly higher than those of *Ma*FAR/*Sc*WS in wild-type *Camelina* (Fig. 6) [15]. In addition, it should be noted that for the analysis of the MaFAR/ScWS-HO lines, T2-plants were used that were producing relatively high amounts of wax esters. However, these lines were not homozygous and may contain more than one insert of the transgenes. Therefore, the number of inserts of the relevant transgenes may differ in the next generation that was analyzed for wax ester content as shown in Fig. 5.

The increase in oleyl oleate in the high oleate background in *Camelina* was less pronounced than that observed for *Ma*FAR/*Ab*WSD1 in the Arabidopsis *fad2 fae1* double mutant, as there were still around 9 mol% 18:1/20:1 and 7 mol% 20:1/20:1 produced by *Ma*FAR/*Sc*WS-HO. More C_18:1_ alcohol was incorporated into wax esters by *Ma*FAR/*Sc*WS-HO (60 mol%) in comparison to less than 40 mol% of *Ma*FAR/*Sc*WS in wild-type *Camelina* (Additional file 8: Figure S4). Thus, the high specificity of MaFAR to C_18:1_ acyl-CoA and of *Sc*WS to very long-chain substrates is reflected in these results. However, an accumulation of only 40 mol% of oleyl oleate by *Ma*FAR/*Sc*WS in the high oleate *Camelina* background is in contrast to the production of the previously observed 60 mol% in high oleate Arabidopsis by the same enzyme combination (Fig. 6) [15]. An explanation for the lower oleyl oleate production might be that although the HO *Camelina* line contains up to 70% oleic acid in its seed oil, the amount of oleic acid is still lower than that of the Arabidopsis *fad2 fae1* double mutant [24, 34]. Therefore, for the formation of oleyl oleate in *Camelina* for industrial applications, the level of oleic acid in seed oil of *Camelina* needs to be further increased using a high-efficiency genome-editing tool, such as the CRISPR/Cas9 technology, instead of using RNA_i_ to knockout fatty acid-editing enzymes.

## Conclusions

Even though higher amounts of wax esters in the seed oil of Arabidopsis were not achieved by the seven enzyme combinations compared with the *Ma*FAR/*Sc*WS combination, the accumulation of oleyl oleate was improved in seed oil of both Arabidopsis and *Camelina* by expression of the proper enzyme combinations in a HO background. Meanwhile, the enzymatic activities and substrate specificities of several wax ester synthesis enzymes were investigated in detail in this study. The species of available acyl substrates dominate the compositions of wax esters in the seed oil while the substrate preference of wax ester forming enzymes has little influence.

## Methods

### Materials

Restriction enzymes and DNA-modifying enzymes were obtained from MBI Fermentas. Tripentadecanoin (tri-15:0) as an internal standard of TAG was purchased from Sigma-Aldrich (Taufkirchen, Germany), and heptadecanoyl heptadecanoate (di-17:0) as an internal standard of wax ester was obtained from Nu-Chek-Prep (Elysian, MN). Chloroform, methanol, *n*-hexane was purchased from Baker (USA), and other chemicals were obtained from Sigma-Aldrich.

### Molecular cloning

To generate *Sc*WS-*Ma*FAR fusion protein, the coding DNA sequences of *Sc*WS (Accession number: Q9XGY6) and *Ma*FAR (Accession number: WP_011785687) were individually amplified using respective primers: *Sc*WS-*for*-*BamH*I/*Sc*WS-linker-*rev* and linker-*Ma*FAR-*for*/*Ma*FAR-*rev*-*Xho*I (Additional file 9: Table S5). The resulting amplicons were fused in PCR reaction using primers *Sc*WS-*for*-*BamH*I/*Ma*FAR-*rev*-*Xho*I. The first 20 amino acids (AA) of *Ab*WSD1 (Accession number: Q8GGG1) were codon optimized for Arabidopsis (PCO*Ab*WSD1) by PCR using primers PCO*Ab*WSD1-*for*-*Sal*I/*Ab*WSD1-*rev*-*BamH*I. DNA sequences of *Ab*WSD1 and the first 60 AA of *Mm*AWAT2 (Accession number: Q6E1M8) were amplified individually from the respective DNA templates using following primers: *Ab*WSD1-*for*/*Ab*WSD1-*rev*-*BamH*I and TM*Mm*AWAT2-*for*-*Sal*I/TM*Mm*AWAT2-*rev* (Additional file 9: Table S5). Then, the DNA sequence of TM*Mm*AWAT2-*Ab*WSD1 fusion protein was generated by overlap extension PCR using primers TM*Mm*AWAT2-*for*-*Sal*I/*Ab*WSD1-*rev*-*BamH*I.

### Generation of yeast expression constructs

The ORFs of *Ma*FAR and *Sc*WS were separately ligated into pYETS2/CT as *Kpn*l/*BamH*I fragments. The ORFs of *Sc*WS-*Ma*FAR, *Mm*AWAT2, PCO*Ab*WSD1 and TM*Mm*AWAT2-*Ab*WSD1 were moved as *BamH*I/*Xhol*I fragments, respectively, into pYETS2/CT. For coexpression of *Ma*FAR with *Sc*WS in *S. cerevisiae*, the ORF of *Ma*FAR was moved as *EcoR*I/*Not*I fragment into the mcs1 of pESC-URA, and the ORF of *Sc*WS was moved as *Bamh*I/*Sal*I fragment into the mcs2 of pESC-URA (Additional file 10: Table S6).

### Generation of plant expression constructs

For stable expressions of different enzymatic combinations in seeds of Arabidopsis, plant transformation vectors were generated using Gateway technology (Invitrogen, Carlsbad) as described previously [14]. This resulted in the constructs: *pCAMBIA33.0*-*pβ*-*CONGLYCININ::Sc*WS-*Ma*FAR, *pCAMBIA33.0*-*β*-*CONGLYCININ::Sc*WS-*Ma*FAR*/pOLEOSIN::Sc*WS-*Ma*FAR, *pCAMBIA33.0*-*pβ*–*CONGLYCININ::Sc*WS–*Ma*FAR*/pOLEOSIN::Ma*FAR, *pCAMBIA33.0*-*pNAPIN::Ma*FAR*/pNAPIN::Ab*WSD1, *pCAMBIA33.0*-*pNAPIN::MaFAR/pNAPIN::*PCO*Ab*WSD1*, pCAMBIA33.0*-*pNAPIN::MaFAR/pNAPIN::*TM*Mm*AWAT2-*Ab*WSD1 (Additional file 10: Table S6).

### Yeast expression

*S. cerevisiae* strain H1246 defective in neutral lipid accumulation [35] was used for expression of different wax synthases. Transformation of *S. cerevisiae* was done as described previously [36]. Expression culture with 2% (w/v) galactose was inoculated at 30 °C and shaking at 18 rpm for 48–96 h. When indicated, fatty alcohol (18:0-OH) was dissolved in ethanol and added into the expression culture to a final concentration of 1 mM. The cells were harvested by centrifugation at 4000 rpm for 10 min and used for lipid analysis. The host strain transformed with empty vector (pYES2/CT) was used as a negative control.

### Plant transformation

Arabidopsis plants were transformed by *Agrobacterium*-mediated floral dipping [37]. *Camelina* plants were transformed via *Agrobacterium*-mediated vacuum infiltration of flowers [22]. The resulting T_1_ plants were grown on soil in climate chamber and selected by phosphinothricin treatment. T_2_ seeds harvested from individual T_1_ plants were used for lipid analysis.

### Plant crossing

A high oleate *Camelina* line containing around 65% oleic acid [24] was used as a mother line and crossed with six individual *Ma*FAR/*Sc*WS lines. Seeds of individual heterozygous plants resulting from these crosses were germinated. One of two cotyledons of individual seedlings was cut off, and their wax ester contents were analyzed by TLC. The seedlings with high amounts of wax esters in cotyledons were planted on soil to propagate seeds of next generation.

### Lipid extraction

Lipid extraction from yeast was done following the method of [32]. Cells corresponding to 50 OD_600_ units were harvested by centrifugation at 4000 rpm for 10 min. Cell‐pellets were resuspended in 1 ml methanol and vortexed for 15 min with 0.5 mm glass beads. Then, the samples were extracted by adding 2 ml *n*-hexane and vortexed for another 15 min. The organic phase of each sample was separated from the polar phase by centrifugation, transferred into a new tube and evaporated under nitrogen stream. Sample was finally resolved in 200 µl *n*-hexane for further analysis.

Extraction of seed lipids was done as described previously [4]. 5 mg seeds of Arabidopsis or 10 mg seeds of *Camelina* were used. The seeds were homogenized in an 8 ml screw lid glass tube with 2 ml chloroform: methanol (1:1, v/v). For GC-FID analysis, 100 µg tripentadecanoate (tri-15:0) and 50 µg heptadecanoyl heptadecanoate (di-17:0) internal standards were added. For nano-ESI–MS/MS analysis, 5 nmol di-17:0 was added. Samples were shaken at 4 °C for 20 min, and extracted with 1 ml *n*-hexane: diethyl ether: glacial acetic acid (65:35:1, v/v/v) by shaking at 4 °C for 10 min. After centrifugation at 1500 rpm for 5 min, the upper phase was collected, and the samples were reextracted with 1 ml *n*–hexane. The organic phases from both extraction steps were combined and dried under nitrogen streaming. The lipid extract was dissolved in 100 μl chloroform for further analysis.

### TLC separation of wax esters and TAGs

Separation of wax esters and TAGs was achieved by TLC. 50 µl yeast lipid extract or 40 µl seed lipid extract was spotted on F60 silica gel glass plate (Merck, Germany), and developed with hexane: diethyl ether: acetic acid (80:20:1, v/v/v) as a running solvent. The separated bands of lipids were visualized by incubating dry TLC plates in CuSO_4_ solution and heating at 190 °C. For GC-FID or nano-ESI–MS/MS analysis, the TLC plate was sprayed with 8-anilino-1-naphthalenesulfonic acid (0.2%, w/v) after development. The lipid bands were marked under UV light and scraped out from the plate. The silica was extracted twice with 1 ml *n*-hexane. After centrifugation, the supernatants were combined and evaporated under nitrogen streaming. For GC-FID analysis, the wax ester and TAG fractions were used for acidic methanolysis process. For nano-ESI–MS/MS analysis, the wax ester fractions were dissolved in 2 ml methanol: chloroform (2:1, v/v) containing 5 mM ammonium acetate.

### GC-FID analysis

For quantification of wax esters and TAGs by GC, acidic methanolysis of wax ester and TAG fractions was performed [38]. The detection of the fatty acid methyl esters and the silylated fatty alcohols was done as described previously [14]. *N, O*-Bis (trimethylsilyl) trifluoroacetamide was mixed with the sample in a ratio of 1:2 (v/v), to separate more efficiently the signals of fatty acid methyl esters and alcohols. As steryl esters are also present in the wax ester samples, the fatty acid profiles of the wax ester fraction of wild-type plants were prepared and subtracted from the corresponding profiles of transgenic plants.

### Nano-ESI–MS/MS analysis

The molecular species of wax esters were measured by nano‐ESI‐MS/MS according to the protocol by [4]. 10 µl wax ester extract was directly subjected to nano‐ESI using a chip ion source (TriVersa NanoMate; Advion BioSciences, USA). The analysis was performed using an Applied Biosystems 3200 hybrid triple quadrupole/linear ion trap mass spectrometer (ABSciex, Germany). The peak intensities of 485 MRM transitions were collected, corresponding to wax ester molecular species with even chained acyl moieties of C_16_–C_24_ containing 0–3 double bonds and C_26_ with 0–1 double bond. To correct the false-positive signals from detector noise or detection of sterol esters, the molar amounts of each MRM transition were also detected in wild-type plants, and subtracted from the molar amounts detected in transgenic plants.

## Additional files

**Additional file 1: Figure S1.** Accumulation of neutral lipids in *S. cerevisiae*. a Cells were transformed with empty vector, *Ma*FAR, *Sc*WS, *Sc*WS-*Ma*FAR fusion protein, *Ma*FAR/*Sc*WS co-expression. b Cells were transformed with empty vector, *Mm*AWAT2, PCO*Ab*WSD1 and TM*Mm*AWAT2-*Ab*WSD1. The host strain with empty vector was used as a negative control. + yeast cells were supplied with fatty alcohol (18:1-OH). − yeast cells were not supplied with fatty alcohol. Yeast were cultivated for 3 days, before the total lipids were extracted from cells corresponding to 50 OD_600_ units. Lipid extractions were analyzed by TLC. Bands of wax ester (WE), triacylglycerol (TAG), free fatty acid (FA) and fatty alcohol (FA-OH) are indicated. Data is representative for two experiments yielding the same results.

**Additional file 2: Table S1.** Raw data obtained by GC-FID to quantify the wax ester content in seeds of transgenic Arabidopsis transformed with *Ma*FAR/*Sc*WS, *Sc*WS-*Ma*FAR, *Sc*WS-*Ma*FAR/*Sc*WS-*Ma*FAR, *Sc*WS-*Ma*FAR/*Ma*FAR, *Ma*FAR/*Ab*WSD1, *Ma*FAR/PCO*Ab*WSD1, *Ma*FAR/TM*Mm*AWAT2-*Ab*WSD1 and *Ma*FAR/*Ma*WS2 shown in Figs. 1 and 3.

**Additional file 3: Table S2.** Raw data obtained by ESI-MS/MS to determine the molecular species of wax esters in seeds of transgenic Arabidopsis transformed with *Ma*FAR/*Ab*WSD1 shown in Fig. 4.

**Additional file 4: Table S3.** Raw data obtained by GC-FID to quantify the contents of wax esters in seeds of *Camelina Ma*FAR/*Sc*WS and High Oleic crosses shown in Fig. 5.

**Additional file 5: Table S4.** Calculation process of ESI-MS/MS data to determine the molecular species of wax esters in seeds of *Camelina Ma*FAR/*Sc*WS and High Oleic crosses shown in Fig. 6.

**Additional file 6: Figure S2.** Three-day seedlings of wild-type *Camelina*, *Ma*FAR/*Sc*WS lines and L9 *Ma*FAR/*Sc*WS and High Oleic cross. The seedlings of transgenic lines producing high levels of wax esters have white cotyledons and are delayed in development in the first 2 weeks.

**Additional file 7: Figure S3.** Codon usage frequency values of *Ma*FAR and *Sc*WS upon expression in *S. cerevisiae*. a *Ma*FAR was optimized for *E. coli* and the photo shows the 151–350 amino acids of *Ma*FAR. b The photo shows the 151–350 amino acids of *Sc*WS. Values were determined using the graphical codon usage analyzer online tool.

**Additional file 8: Figure S4.** Alcohol and acyl moieties of wax esters in seeds of *Camelina* with *Ma*FAR/*Sc*WS and six *Ma*FAR/*Sc*WS and High Oleic crosses. The *Ma*FAR/*Sc*WS line (purple bar), L4 *Ma*FAR/*Sc*WS and High Oleic cross (orange bar), L5 *Ma*FAR/*Sc*WS and High Oleic cross (blue bar), L9 *Ma*FAR/*Sc*WS and High Oleic cross (red bar), L13 *Ma*FAR/*Sc*WS and High Oleic cross (green bar), L25 *Ma*FAR/*Sc*WS and High Oleic cross (yellow bar), L26 *Ma*FAR/*Sc*WS and High Oleic cross (light blue bar) are shown. a Relative abundance of alcohol moieties in mol%. b Relative abundance of acyl moieties in mol%. The data shown represent an average of three individual transgenic lines for each construct with two extraction replicates measured by GC-FID, and raw data shown in Additional file 4: Table S3.

**Additional file 9: Table S5.** List of primers used in this study for amplifying DNA sequences of wax ester synthesis enzymes.

**Additional file 10: Table S6.** List of DNA constructs used in this study for expressing wax ester synthesis enzymes.

## Abbreviations

AA: amino acids
ACP: acyl carrier protein
*Ab*WSD1: bifunctional waxsynthase/acyl-CoA:diacylglycerol *O*-acyltransferase from *Acinetobactor baylyi* ADP1
CoA: coenzyme A
DGAT: acyl-CoA:diacylglycerol
ER: endoplasmic reticulum
ESI‐MS/MS: electrospray ionization mass spectrometry
HO: high oleic acid
*Ma*FAR: fatty acyl-CoA reductase from *Marinobacter aquaeolei*
*Ma*FAR/*Sc*WS–HO: HO lines crossed with lines expressing *Ma*FAR and *Sc*WS
*Ma*WS: wax synthase from *Marinobacter aquaeolei* VT8
*Mm*AWAT2: wax synthase from *Mus musculus*
TLC: thin layer chromatography
TM: transmembrane domain
WS: wax synthase
*Sc*WS: wax synthase from *Simmondsia chinensis*
