# Supplementary figures and images for: High-level accumulation of oleyl oleate in plant seed oil by abundant supply of oleic acid substrates to efficient wax ester synthesis enzymes
Source: Biotechnol Biofuels. 2018 Mar 1;11:53. doi: 10.1186/s13068-018-1057-4 (PMC5831613; doi:10.1186/s13068-018-1057-4)

a

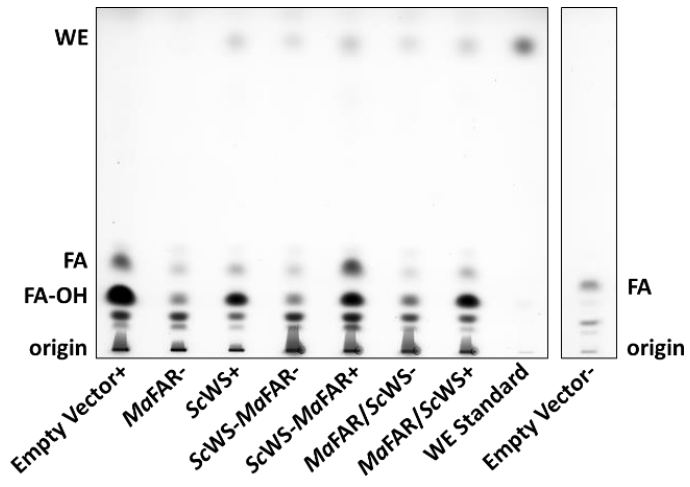

b

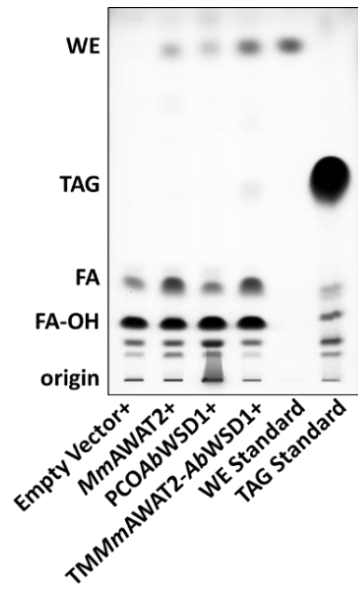

Supplement: Supplementary file 1 — Additional file 1: Figure S1. Accumulation of neutral lipids in S. cerevisiae. a Cells were transformed with empty vector, MaFAR, ScWS, ScWS-MaFAR fusion protein, MaFAR/ScWS co-expression. b Cells were transformed with empty vector, MmAWAT2, PCOAbWSD1 and TMMmAWAT2-AbWSD1. The host strain with empty vector was used as a negative control. + yeast cells were supplied with fatty alcohol (18:1-OH). − yeast cells were not supplied with fatty alcohol. Yeast were cultivated for 3 days, before the total lipids were extracted from cells corresponding to 50 OD600 units. Lipid extractions were analyzed by TLC. Bands of wax ester (WE), triacylglycerol (TAG), free fatty acid (FA) and fatty alcohol (FA-OH) are indicated. Data is representative for two experiments yielding the same results. [file 13068_2018_1057_MOESM1_ESM.pdf]

**Wild type**

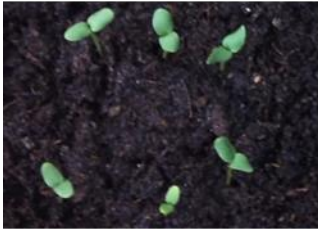

**MaFAR/ScWS**

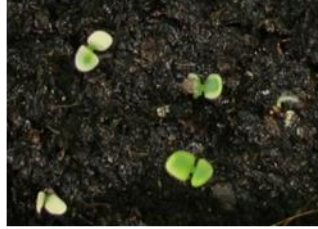

**L9 MS & HO cross**

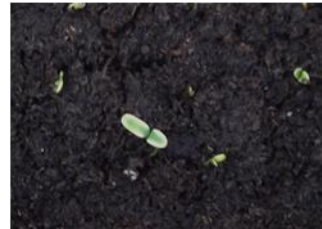

Supplement: Supplementary file 6 — Additional file 6: Figure S2. Three-day seedlings of wild-type Camelina, MaFAR/ScWS lines and L9 MaFAR/ScWS and High Oleic cross. The seedlings of transgenic lines producing high levels of wax esters have white cotyledons and are delayed in development in the first 2 weeks. [file 13068_2018_1057_MOESM6_ESM.pdf]

a

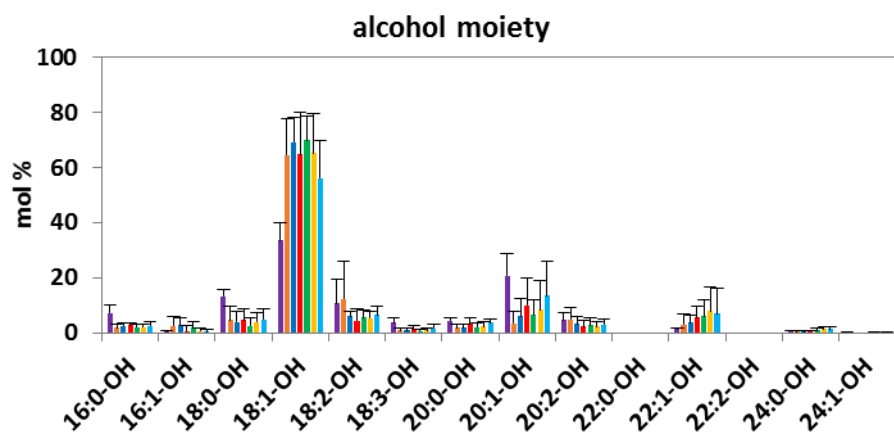

b

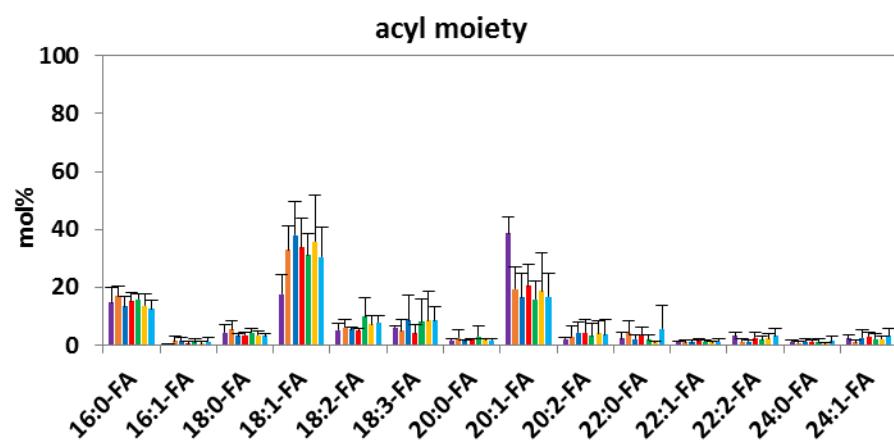

Supplement: Supplementary file 8 — Additional file 8: Figure S4. Alcohol and acyl moieties of wax esters in seeds of Camelina with MaFAR/ScWS and six MaFAR/ScWS and High Oleic crosses. The MaFAR/ScWS line (purple bar), L4 MaFAR/ScWS and High Oleic cross (orange bar), L5 MaFAR/ScWS and High Oleic cross (blue bar), L9 MaFAR/ScWS and High Oleic cross (red bar), L13 MaFAR/ScWS and High Oleic cross (green bar), L25 MaFAR/ScWS and High Oleic cross (yellow bar), L26 MaFAR/ScWS and High Oleic cross (light blue bar) are shown. a Relative abundance of alcohol moieties in mol%. b Relative abundance of acyl moieties in mol%. The data shown represent an average of three individual transgenic lines for each construct with two extraction replicates measured by GC-FID, and raw data shown in Additional file 4: Table S3. [file 13068_2018_1057_MOESM8_ESM.pdf]
