# Supplementary material for: High-level accumulation of oleyl oleate in plant seed oil by abundant supply of oleic acid substrates to efficient wax ester synthesis enzymes
Source: Biotechnol Biofuels. 2018 Mar 1;11:53. doi: 10.1186/s13068-018-1057-4 (PMC5831613; doi:10.1186/s13068-018-1057-4)

a

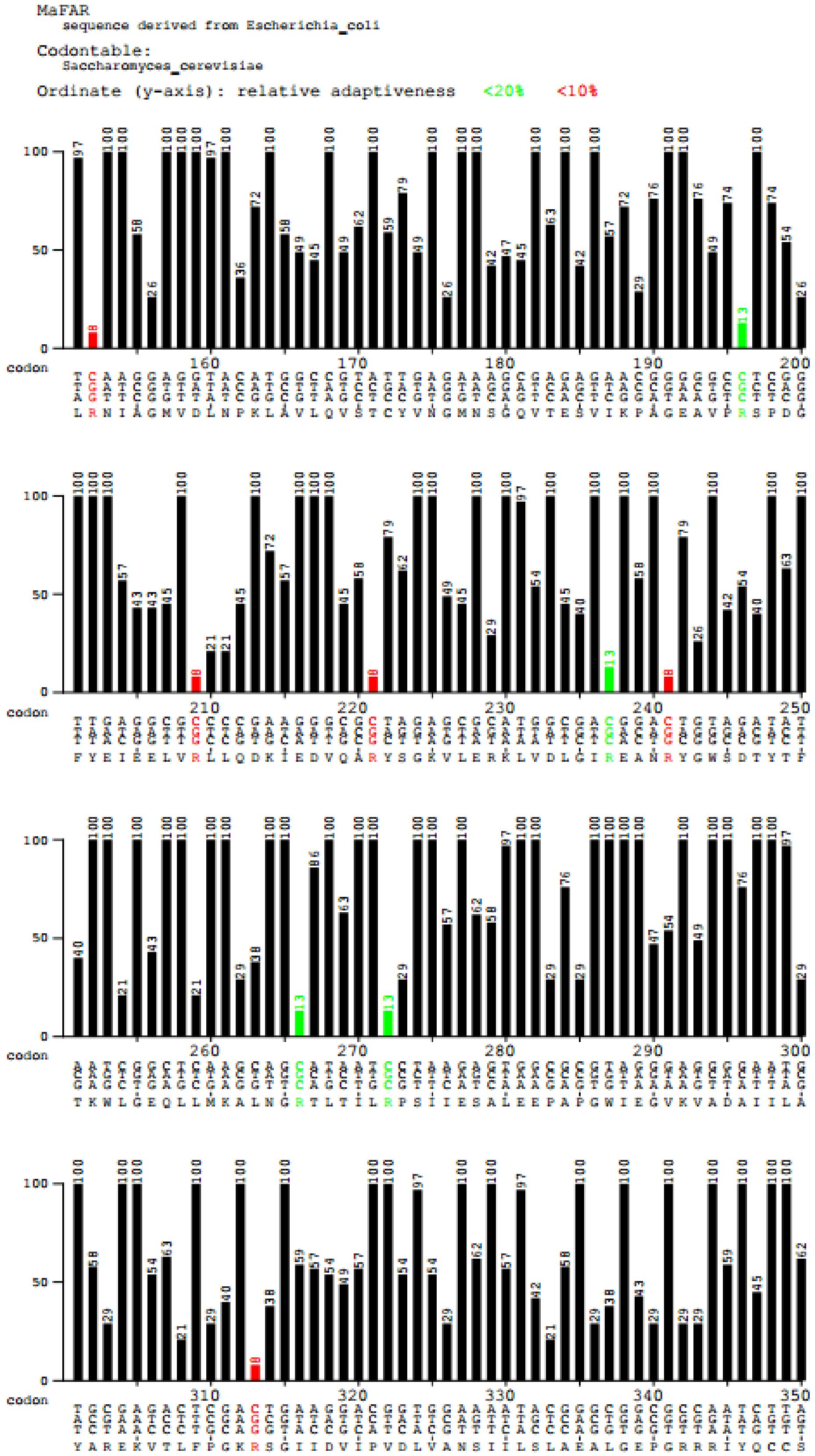

MaFAR
sequence derived from Escherichia_coli
Codontable:
Saccharomyces_cerevisiae
Ordinate (y-axis): relative adaptiveness <20% <10%
codon

b

ScWS
sequence derived from Arabidopsis_thaliana

Codontable:
Saccharomyces_cerevisiae

Ordinate (y-axis): relative adaptiveness <20% <10%

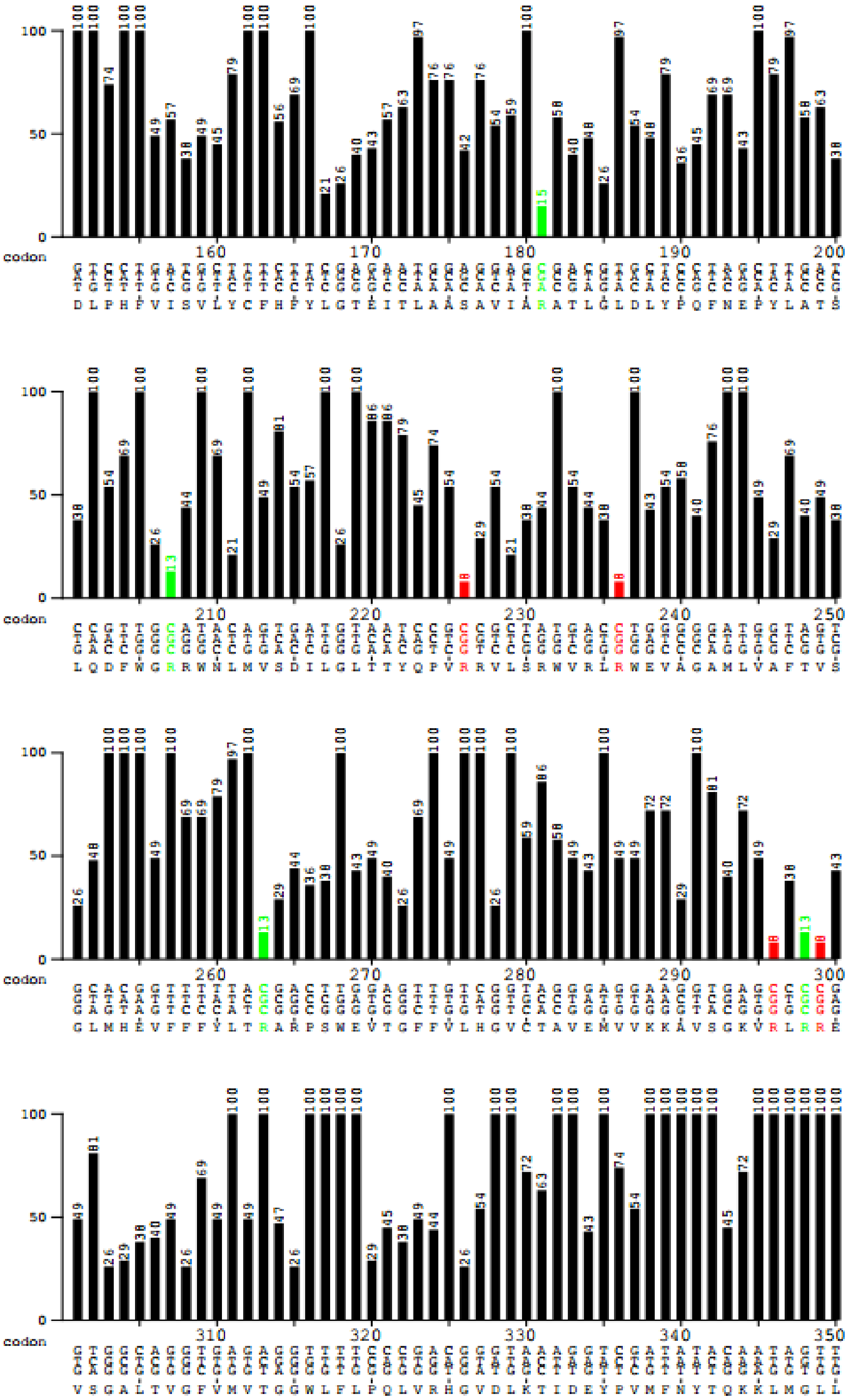

Supplement: Supplementary file 7 — Additional file 7: Figure S3. Codon usage frequency values of MaFAR and ScWS upon expression in S. cerevisiae. a MaFAR was optimized for E. coli and the photo shows the 151–350 amino acids of MaFAR. b The photo shows the 151–350 amino acids of ScWS. Values were determined using the graphical codon usage analyzer online tool. [file 13068_2018_1057_MOESM7_ESM.pdf]
